# Promoter recruitment drives the emergence of proto-genes in a long-term evolution experiment with *Escherichia coli*

**DOI:** 10.1371/journal.pbio.3002418

**Published:** 2024-05-07

**Authors:** Md. Hassan uz-Zaman, Simon D’Alton, Jeffrey E. Barrick, Howard Ochman

**Affiliations:** Department of Molecular Biosciences, University of Texas at Austin, Austin, Texas, United States of America; Trinity College Dublin: The University of Dublin Trinity College, IRELAND

## Abstract

The phenomenon of de novo gene birth—the emergence of genes from non-genic sequences—has received considerable attention due to the widespread occurrence of genes that are unique to particular species or genomes. Most instances of de novo gene birth have been recognized through comparative analyses of genome sequences in eukaryotes, despite the abundance of novel, lineage-specific genes in bacteria and the relative ease with which bacteria can be studied in an experimental context. Here, we explore the genetic record of the *Escherichia coli* long-term evolution experiment (LTEE) for changes indicative of “proto-genic” phases of new gene birth in which non-genic sequences evolve stable transcription and/or translation. Over the time span of the LTEE, non-genic regions are frequently transcribed, translated and differentially expressed, with levels of transcription across low-expressed regions increasing in later generations of the experiment. Proto-genes formed downstream of new mutations result either from insertion element activity or chromosomal translocations that fused preexisting regulatory sequences to regions that were not expressed in the LTEE ancestor. Additionally, we identified instances of proto-gene emergence in which a previously unexpressed sequence was transcribed after formation of an upstream promoter, although such cases were rare compared to those caused by recruitment of preexisting promoters. Tracing the origin of the causative mutations, we discovered that most occurred early in the history of the LTEE, often within the first 20,000 generations, and became fixed soon after emergence. Our findings show that proto-genes emerge frequently within evolving populations, can persist stably, and can serve as potential substrates for new gene formation.

## Abstract 2

De novo gene birth from non-genic sequence has received considerable attention due to the widespread occurrence of genes that are unique to particular species. This study shows that within the short evolutionary timescale of the iconic E. coli LTEE experiment, the co-option of promoters, principally from transposable elements, serves as the major source of raw material for new genes.

## Introduction

New genes are thought to originate mostly through a process of duplication and divergence, in which copies of already existing genes are repurposed to serve new functions [1–3]. This process, however, requires the presence of preexisting genes and does not address how genes that serve as substrates for duplication and divergence originally arose. The de novo origin of genes from non-genic sequences, i.e., regions other than existing protein-coding or RNA genes, began receiving consideration in the 1990s [4]. The potential for new genes to emerge in this way was reinforced by both the functional characterization of putative de novo genes [5,6] and the widespread identification of lineage-specific genes in virtually every species [7–12]. Furthermore, transcriptome surveys established that non-genic regions of the genome are continually subject to stochastic transcription and translation [13–16], such that new genes could arise if a non-genic sequence manifests a function and evolves more stable expression.

De novo gene formation, which has been studied extensively in eukaryotes [17] and viruses [18,19], appears also to contribute to bacterial evolution. A substantial fraction of the gene repertoires of bacterial species are lineage-specific [20,21] and such genes often show no clear homologs, indicating that they may not have originated by duplication and divergence [20,22] or horizontal gene transfer [23,24]. Moreover, some lineage-specific genes have been traced to noncoding sequences, suggesting the possibility of de novo emergence [25]. Although bacterial genomes contain little intergenic DNA, both strands of their genomes are transcribed pervasively [26,27], and a number of bacterial genes have been found to be derived from the opposite strand or within shifted reading frames of existing genes [28–30].

**Fig 1:**
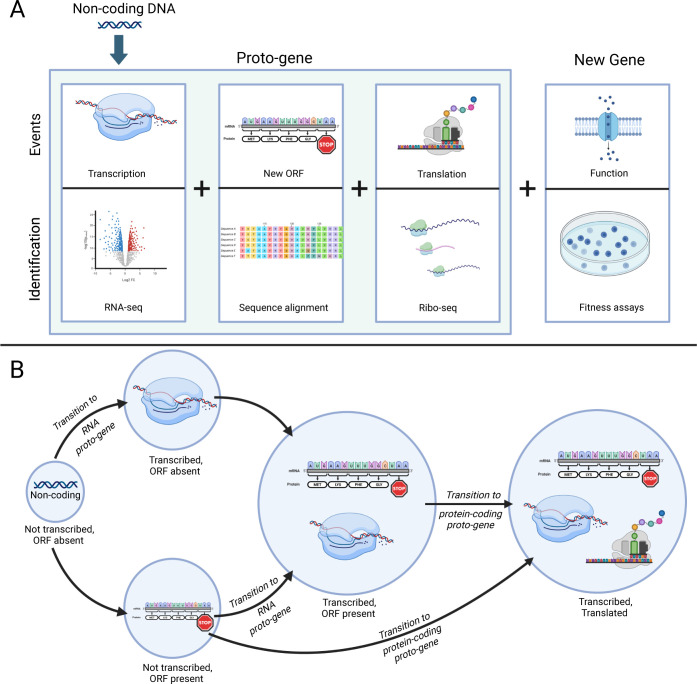
Stages of proto-gene emergence. (A) Events required for the birth of proto-genes (shaded area) and new genes, and examples of experimental methods that can identify these events. Modified and expanded from [31]. (B) Ways in which a noncoding region can transition into a proto-gene. Created with BioRender.

De novo emergence of a new gene can be conceptualized as the co-occurrence of the following: (i) transcription of the sequence, and in the case of protein-coding genes; (ii) translation of an open reading frame (ORF) within the transcript; and (iii) appearance of a beneficial function [31,32] (Fig 1A). Following the terminology offered in [32], noncoding sequences arriving at either of the 2 intermediate stages on the way to becoming functional genes are considered “proto-genes,” represented by the transition of ancestrally silent sequences to a state of transcription and potentially also translation (Fig 1B).

To date, most de novo genes have been recognized through retrospective and comparative analyses [33,34]; however, unicellular eukaryotes and bacteria offer the opportunity to experimentally investigate gene birth on account of their short generation times and potential for rapid evolution. Here, we use genome sequencing, transcriptomics (RNA-seq), and ribosome profiling (Ribo-seq) data from the *Escherichia coli* long-term evolution experiment (LTEE) [35–37] to directly detect the emergence of proto-gene transcription and translation associated with new mutations. We demonstrate that within the timescale and environment of the LTEE, proto-genes, as represented by novel transcripts and peptides, emerge most frequently via recruiting existing promoters, can persist in subsequent generations once they arise and reach fixation within the population.

## Results

### Non-genic transcription and translation are frequent in the LTEE

**Fig 2:**
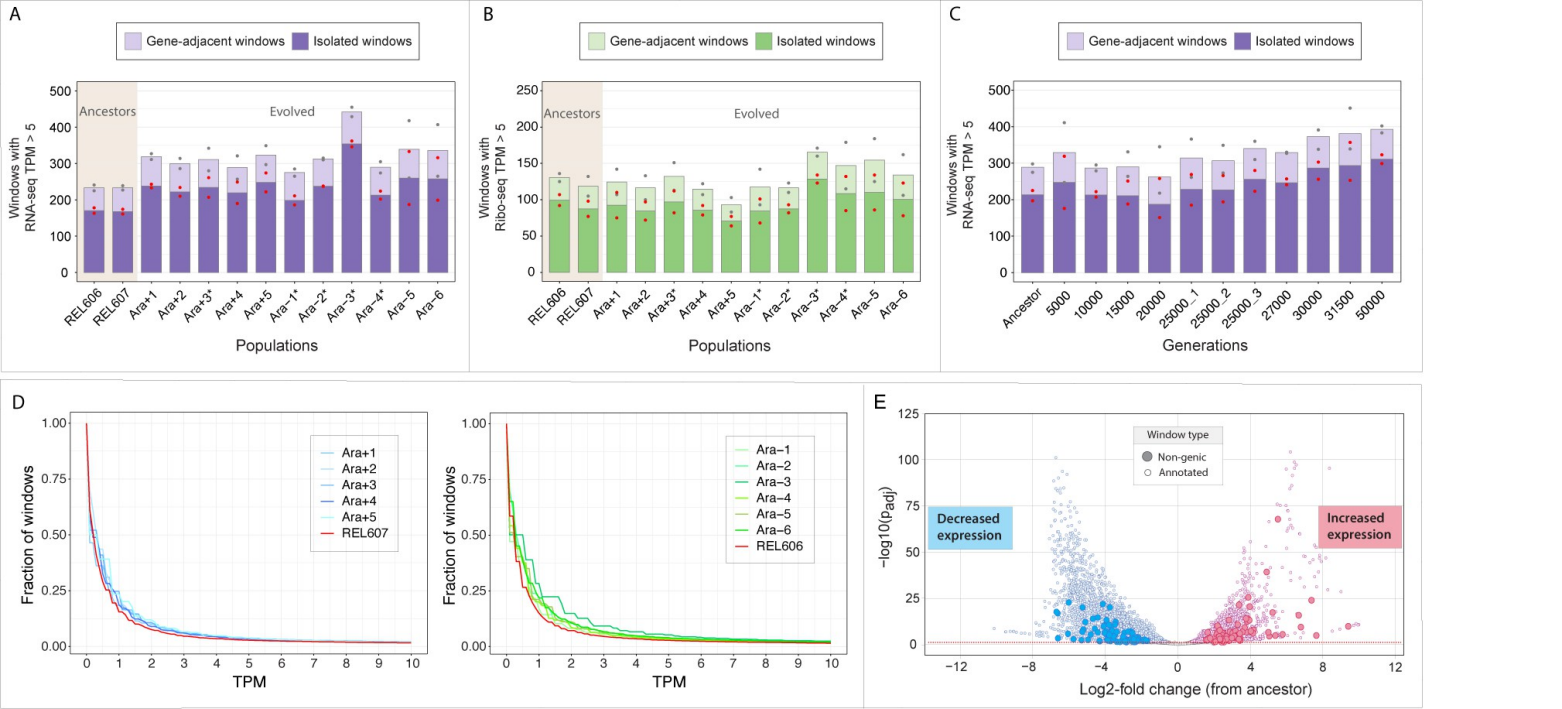
Expression of non-genic regions. Numbers of non-genic windows in ancestral and evolved populations with normalized read counts expressed in average number of TPM: (A) RNA-seq, 50,000 generations; (B) Ribo-seq, 50,000 generations, and (C) RNA-seq, time series. Bar height represents the average TPM between 2 (50,000-generation) or 3 (time series) replicates. Windows overlapping with the 300 bp upstream or the 100 bp downstream of an annotated gene are labeled as “Gene-adjacent windows,” with the remaining labeled as “Isolated windows.” Red and gray points represent highest and lowest TPM values among replicates in Gene-adjacent and Isolated windows, respectively. (D) Cumulative frequency distribution of windows passing different TPM cutoffs in the 50,000-generation dataset. Values for the 2 ancestral strains are depicted in red. Cutoffs are set at 0.1 TPM intervals. (E) Volcano plot of all windows whose expression changed between ancestral and evolved populations at generation 50,000. Dotted red line denotes *p*_*adj*_ = 0.05. The data underlying this figure can be found in https://zenodo.org/records/10980486.

To assess the overall extent of transcription and translation occurring in non-genic regions in the LTEE, we surveyed genome-wide expression levels in 400-bp sliding windows along both strands of the genome in RNA-seq and Ribo-seq datasets. Of 18,745 such windows in the LTEE ancestor genome, 9,987 overlapped annotated genes on the same strand, 8,599 on the opposite strand (antisense), and 159 were intergenic (S1 File). Transcription and translation in non-genic regions, which include both antisense and intergenic windows, were detected in all lines and time points surveyed (Figs 2A–2C and S2 and S1 File).

We first examined RNA-seq data of clones isolated from 11 LTEE lines at 50,000 generations (Fig 2A). In this dataset, 94.9% of windows overlapping annotated protein-coding or RNA genes and 63.7% of the non-genic windows were transcribed in at least 1 clone at a relaxed threshold of 1 transcript per million reads (TPM). When raising the threshold to >5 TPM, 73.8% annotated and only 9.8% of non-genic windows met this more stringent cutoff. After eliminating regions located within 300-bp upstream or 100-bp downstream of annotated genes (to account for both transcription initiation before the start codon and readthrough), this fraction fell to 8.8% for non-genic windows (S1 File).

Focusing next on Ribo-seq data from the same clones (Fig 2B), whereas a similar number of annotated windows were translated at the >1 TPM level as were transcribed (92.9% versus 94.9%), the fraction of translated non-genic windows fell to 37.2%. At the more stringent cutoff, only 4.1% of non-genic windows contained reads >5 TPM compared to 72.1% of annotated windows. Among the more highly transcribed (>5 TPM) non-genic windows not adjacent to annotated genes (termed “isolated windows” in Fig 2), 25.1% met the >5 TPM Ribo-seq cutoff, whereas a vast majority (94.0%) of annotated windows transcribed at this level showed evidence of translation. Furthermore, a total of 233 non-genic (183 isolated) windows experienced significant changes in transcription at 50,000 generations when compared to their ancestors (*p*_*adj*_ < 0.05, Wald test *p*-value adjusted by Benjamini–Hochberg method) (Fig 2E); however, none was differentially translated after accounting for the transcriptional change. Taken together, the extent of transcription, translation, and differential expression in the non-genic regions of evolved genomes indicates the presence of substantial raw material for new gene formation in the LTEE.

### Non-genic transcription increases in the course of LTEE

Ancestral and evolved clones averaged 269.5 and 394.2 non-genic windows transcribed at >5 TPM, respectively (Fig 2A and S1 File). Most evolved clones had significantly more transcribed windows than their ancestors, and this trend held even when considering a lower transcription threshold (1 TPM), a smaller window size (100 bp), for windows with or without overlap with annotated genes, and for clones from populations that evolved elevated mutation rates or those that maintained the low ancestral rate (paired two-sample *t* tests of data for evolved clone versus its ancestor, *p* < 0.001) (S1 File). Unlike the case for transcription, translation across these non-genic windows was unchanged for most evolved clones relative to their ancestors (Fig 2B).

In a separate time series dataset generated from 1 population (Ara−3), significant changes in the number of transcribed non-genic windows were observed only in samples from later generations, specifically around generation 30,000 (Fig 2C and S1 File). Interestingly, this trend disappears when considering very low TPM thresholds (<0.3 TPM) (Fig 2D), suggesting that completely unexpressed regions do not generally gain transcription, whereas regions with minimal levels of transcription start being expressed at higher rates in the later time points.

### New mutations coincide with novel transcription

**Fig 3:**
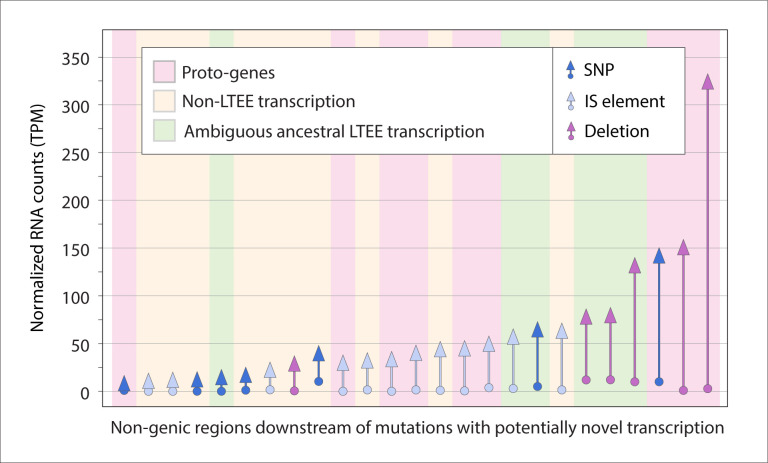
Mutations leading to novel transcription in LTEE populations. Arrows along *x*-axis represent regions displaying significant increases in expression, colored according to mutation type. Arrow lengths show differences in TPM between the ancestral and evolved states, with regions sorted according to the magnitude of change. Background shading represents different classes of proto-gene candidates. (Note that the figure includes 19 unambiguous proto-gene candidates and the 6 transcripts with ambiguous transcription in the ancestor.) The data underlying this figure can be found in https://zenodo.org/records/10980486.

Non-genic transcription gains that result from mutations leading to promoter acquisition are more likely to persist and potentially be co-opted as new genes than non-heritable noise in transcription [38]. We therefore focused on transcription increases coinciding with the appearance of new mutations to explore proto-gene emergence. We first identified all cases in which a non-genic region immediately downstream of a new mutation experienced an increase in transcription compared to the ancestor. In the 50,000-generation dataset, there were 63 statistically significant cases (*p*_*adj*_ < 0.05, see Methods). To concentrate on cases of novel transcription, we visually inspected RNA-seq coverage plots to eliminate candidates that were transcribed at any level in the ancestor (S2 File). This procedure yielded 19 unambiguous proto-gene candidates with expression levels that increased by as much as 2 orders of magnitude (Fig 3; which also includes 6 ambiguous candidates). Implementing the same pipeline on the Ribo-seq data, we detected only 1 region of novel translation associated with a mutation (S3 File).

**Fig 4:**
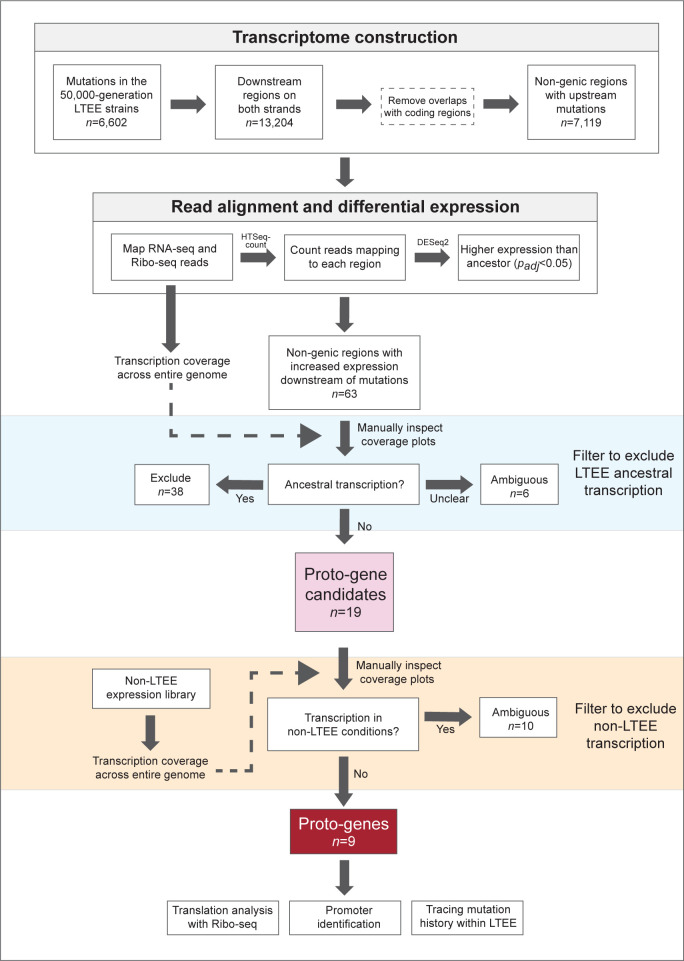
Workflow for detecting proto-genes.

Since the regions with novel transcription could represent unannotated genes that were initially silent under the unvarying experimental conditions of the LTEE, we surveyed datasets that measured gene expression in the LTEE ancestor across a wide range of environmental conditions and growth phases [39,40]. After eliminating the candidates that exhibited transcription in any of these conditions (“non-LTEE transcription” in Fig 4), a final set of 9 proto-gene candidates remained in the samples from the 50,000-generation time point (Tables 1, S2, and S5, and S3 Fig).

**Table 1:** List of proto-genes identified in this study.

Proto-gene ID	Population	Start position in ancestral genome	Mutation type	Dataset^a^	Cause of transcription/translation gain	Corresponding figure
Ara−2_731_SNP	Ara−2	3041423	SNP	50K	Unknown	6A
Ara−2_610_SNP	Ara−2	2544773	SNP	50K	Unknown	S2 File
Ara−3_547_MOB	Ara−3	3015771	IS*150*	50K, TS	Promoter in insertion sequence	5, 8A
Ara−6_59_DEL	Ara−6	3289782	7849-bp deletion	50K	Large deletion brings a non-genic region immediately downstream of a promoter	7
Ara−6_24_MOB	Ara−6	1515685	IS*150*	50K	Promoter in insertion sequence	5
Ara+1_99_MOB	Ara+1	3697154	IS*150*	50K	Promoter in insertion sequence	5
Ara+1_94_DEL	Ara+1	3482398	25-bp deletion	50K	Deletion leads to new promoter formation	6B
Ara+5_65_MOB, Ara−3_4110237_MOB^b^	Ara+5, Ara−3	4110237	IS*150*	50K, TS	Promoter in insertion sequence	5, 8C
Ara+5_30_MOB	Ara+5	2196654	IS*150*	50K	Promoter in insertion sequence	5

^a^50K and TS refer to the 50,000-generation and Ara−3 time series datasets, respectively.

^b^The same proto-gene emerged twice in 2 different populations.

To confirm that these 9 proto-genes are unique to the LTEE, we searched their sequences against a catalogue of both annotated and non-annotated transcripts assembled for *E*. *coli* K-12 MG1655 [41] consisting of 9,581 transcripts extracted from 3,376 RNA-seq experiments. All but one (Ara−3_547_MOB) of the 9 proto-gene sequences were present in the K-12 MG1655 genome, but none matched any annotated or unannotated transcript. The similarity between the LTEE ancestor and K-12 MG1655 (ANI = 99% [42]) indicates that expression of these sequences in *E*. *coli* prior to the initiation of the LTEE was unlikely, further supporting that they are proto-genes that emerged during laboratory evolution (Table 1).

### Insertion sequences frequently contribute to novel transcription

**Fig 5:**
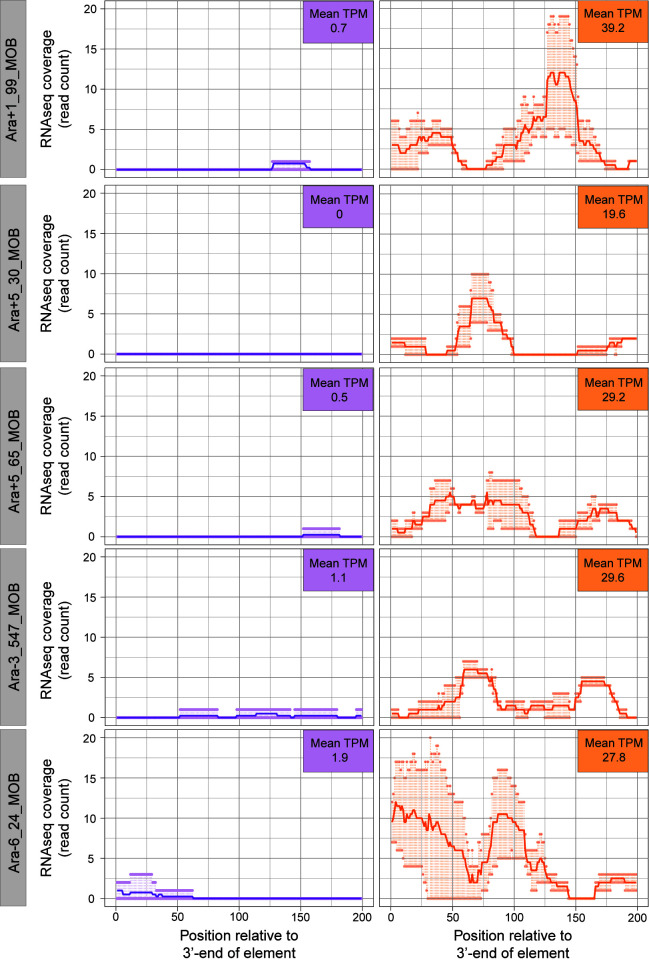
Novel transcription downstream of IS*150* insertions at generation 50,000. The 5 proto-genes formed by this mechanism are shown. Purple and orange lines denote ancestral and evolved transcription. Darker lines represent average read count, with points above and below depicting maximum and minimum counts among replicates. The data underlying this figure can be found in https://zenodo.org/records/10980486.

For 5 of the 9 proto-genes, new transcriptional activity could be attributed to the activity of insertion sequence IS*150* (Fig 5), a 1,443-bp transposable element which carries an outward-facing promoter that can trigger downstream transcription. To investigate the generality of this effect, we surveyed all non-genic regions throughout the transcriptome that acquired an upstream IS*150* element by generation 50,000. Of 55 such regions, 12 displayed statistically significant increases in transcription, and in all but one case, the regions were silent in the LTEE ancestor. While these 11 passed the initial filter for proto-gene detection (Fig 4), 6 were excluded from the proto-gene list owing to their ambiguous transcription in the non-LTEE samples. The remaining 43 regions were either transcribed in the ancestor strain or did not show a statistically significant increase, indicating that the presence of an upstream IS*150* promoter alone might not be sufficient to produce novel transcription detectable by our approach. In general, transcription gains mediated by the IS*150* promoter are modest compared to those caused by other types of mutations (Fig 3).

### Contribution of point mutations and small deletions to proto-gene emergence

**Fig 6:**
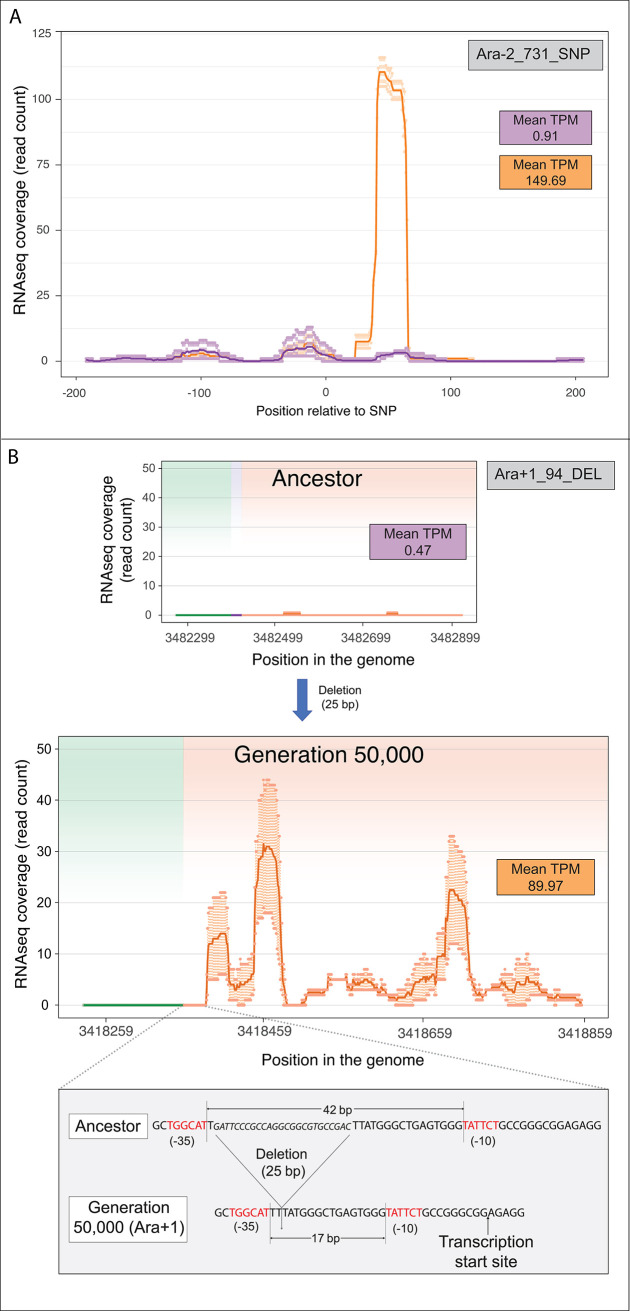
Base substitution- and deletion-associated expression of ancestrally non-transcribed regions. (A) A base substitution in population Ara−2 of the LTEE led to downstream transcription, although no newly formed promoters were detected in this region. Darker lines represent average read count, with points above and below depicting maximum and minimum counts among replicates. Purple and orange lines represent ancestral and evolved transcription, respectively. (B) A deletion of 25 bp in population Ara+1 of the LTEE led to de novo formation of a promoter and downstream expression. Green and orange shading represent regions immediately upstream and downstream of the mutation. The data underlying this figure can be found in https://zenodo.org/records/10980486.

Two of the proto-genes, both in the Ara−2 LTEE population, were associated with upstream single-base substitutions (Fig 6A), although newly formed promoters in the mutated regions were not recognized by promoter prediction tools [43,44]. In another case, in the Ara+1 population, a 25-bp deletion brought 2 preexisting sequences that resemble the −10 and −35 motifs of a σ^70^ promoter into proximity, leading to expression of the proto-gene (Fig 6B). Although base substitutions and small indels comprise the vast majority (95.7%) of the mutations in the LTEE near non-genic regions, these classes of mutations led to novel transcription in only 0.05% of cases, compared to 3% for insertion sequences (S3 Table).

### Emergence of a novel ribosome-associated transcript via a large deletion event

**Fig 7:**
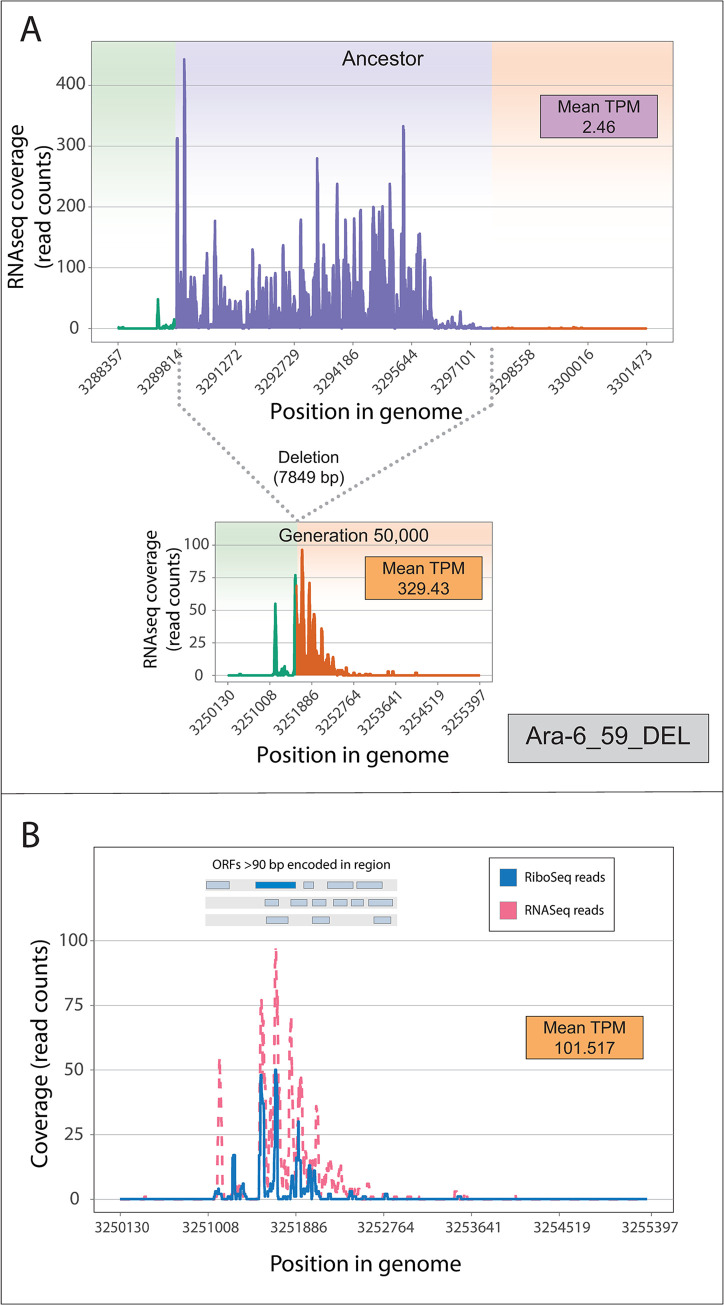
Large deletion-induced transcription and translation of ancestrally non-transcribed regions. (A) 7,849-bp deletion spanning multiple genes (violet) placed a non-transcribed region in proximity of strong promoter (green), leading to expression downstream (orange). (B) Translation of same region in evolved population. Top insert shows positions of ORFs >90 bp within region encoded on transcribed strand (dark blue segment denoting the longest ORF). Inset shows mean TPM Ribo-seq value. The data underlying this figure can be found in https://zenodo.org/records/10980486.

With respect to the expression levels of the proto-genes, the largest increase was elicited by a 7,849-bp deletion that overlapped 6 genes (*gltB*, *gltD*, *yhcG*, *ECB_03080*, *yhcH*, *nanK*) in the Ara−6 line (Fig 7A). This deletion shifted a transcriptionally silent region antisense to *nanK* and *nanE* to a position adjacent to and downstream of the promoter region of the glutamate synthase operon, resulting in new transcription. In the ribosome profiling data from this clone, there are pronounced buildups of reads in the corresponding region (Fig 7B), suggestive of translated ORFs. The longest ORF (117 aa) is homologous to hypothetical proteins in *E*. *coli* and *Shigella sonnei*, indicating that it has gene-like features as recognized by standard prokaryotic annotation pipelines. Since none of the other proto-genes were associated with Ribo-seq reads, this ORF is the only example of a novel ribosome-associated transcript that we identified in the present study.

### Up-regulation is often stable across thousands of generations

**Fig 8:**
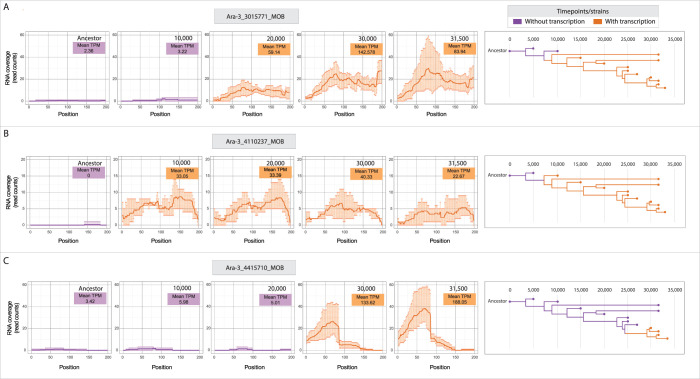
Persistent gains in transcription in Ara−3 population. Left: Levels of transcription in 3 proto-genic regions (A–C) at 5 time points in the LTEE. Darker lines represent average read counts, with points above and below depicting maximum and minimum counts among replicates. Right: Genealogies of time series clones showing the transcription status of corresponding regions at each time point. The data underlying this figure can be found in https://zenodo.org/records/10980486.

If the proto-genes formed by new mutations at generation 50,000 were of recent origin or transitory, it would diminish the likelihood that these newly expressed regions would be co-opted as new genes. Therefore, to investigate the extent to which transcriptional changes forming proto-genes persist in the LTEE lines, we analyzed an RNA-seq dataset generated at different time points of the Ara−3 line using the pipeline described above (Figs 4 and S1). We found 9 candidates with significantly increased expression at any evolved time point, of which 5 showed no transcription in the original LTEE ancestor (S4 File and S2 Table). Two of these 5 were also recognized in the 50,000-generation data for the same line (above), with 1 retained in the final list of proto-genes (Table 1, “Ara−3_547_MOB”). In addition, we detected a new case of mutation-adjacent novel transcription in the time series dataset that was not recognized in the 50,000-generation dataset because its expression change did not reach statistical significance (Table 1, “Ara−3_4110237_MOB”). Interestingly, the same proto-gene arose independently in the Ara+5 line via the same mutation. Overall, these results demonstrate that some newly emerged proto-genes persist in lineages that evolved in the LTEE for tens of thousands of generations (Fig 8).

### Proto-genes can arise rapidly and become widespread in evolving populations

**Fig 9:**
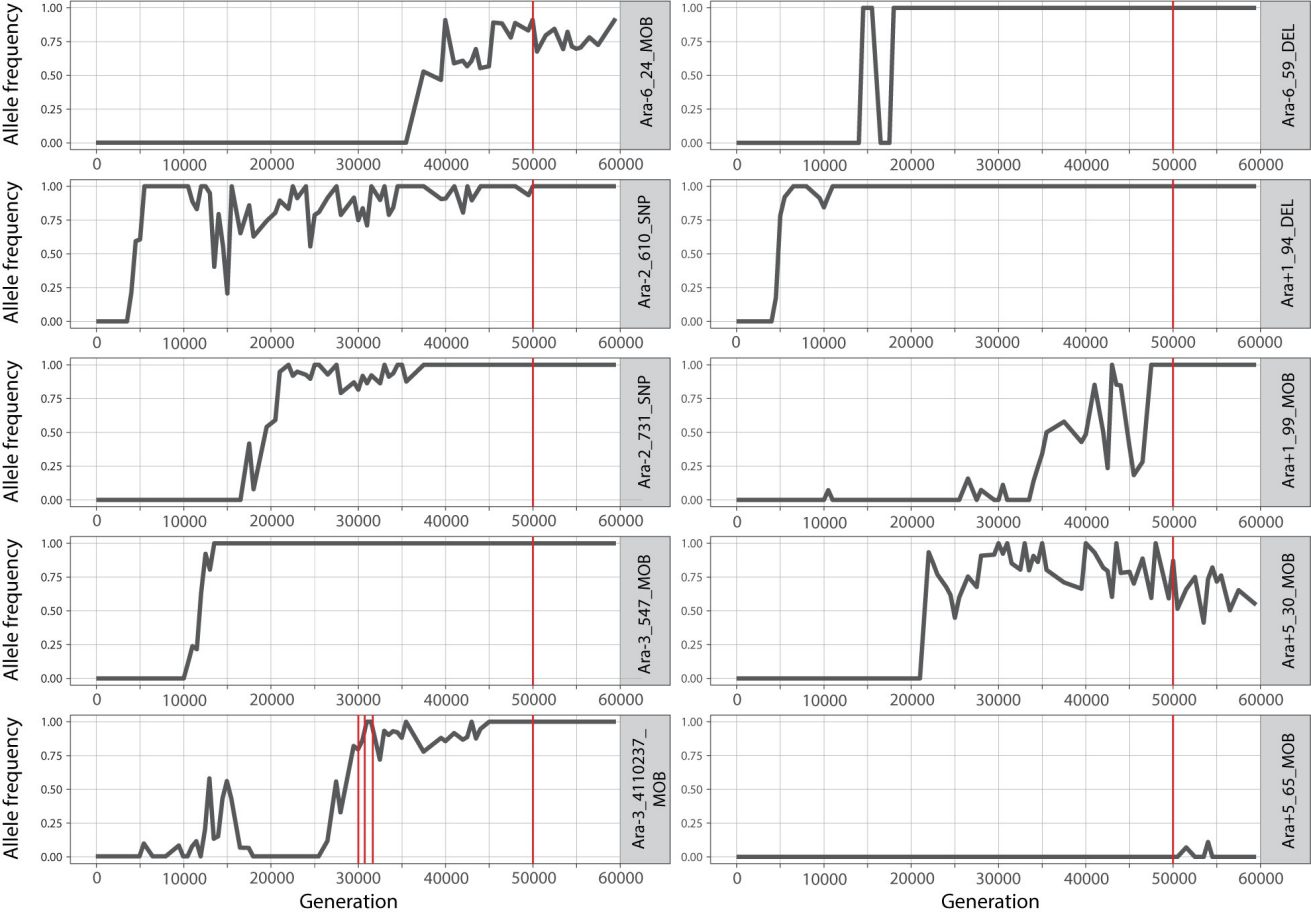
Emergence and allele frequencies of mutations associated with proto-genes. Red lines indicate time points at which transcription was detected. The data underlying this figure can be found in https://zenodo.org/records/10980486.

To further investigate the persistence of proto-genes, we traced the origins of their associated mutations. To this end, we searched for the 10 proto-gene-associated mutations (9 from the 50,000-generation dataset and 1 from the time series) in genomic data generated for the first 60,000-generations of the LTEE [35]. Leveraging this population-sequencing dataset, we inferred the frequency of each mutation across time points, and in all but one case, the mutation was abundant in the population at both earlier and later time points (Fig 9). The 3 proto-genes with the largest increases in transcription (Figs 6 and 7) arose before the 20,000-generation time point and appear to have reached fixation (100% frequency in the population) soon thereafter. The Ara+5_65_MOB mutation, an IS*150* insertion in the Ara+5 line, was not observed at the 50,000-generation time point, and only occurred at low frequencies at 2 subsequent time points. This mutation occurred independently in the Ara−3 line, suggesting the existence of an insertional hotspot in this region.

## Discussion

Within the time-scale of the *E*. *coli* LTEE [35,37], proto-genes have emerged and persisted—some arising in the early stages of the experiment. A primary mechanism by which proto-genes are created is through the acquisition of regulatory sequences that lead to transcription and translation of previously silent regions (Fig 1B). We observed 3 routes by which this occurred: (i) expression induced by new copies of an insertion sequence; (ii) a large deletion that placed an existing promoter upstream of a previously non-transcribed region; and (iii) point mutations and a small deletion that created new promoters.

Transcription from an outward-facing promoter located at the 3′-end of IS*150* [45], due to insertions of new copies of this element, caused over half of the proto-gene emergence events. Insertion sequences have been implicated in regulatory evolution [46,47], and in the LTEE their insertions and deletions account for as many as 50% of total mutations in one population [48,49]. IS*150* is the most actively proliferating element in the LTEE; however, the increases in expression introduced by new IS*150* insertions are generally modest, in most cases failing to reach statistical significance.

The most pronounced case of novel transcription was induced by the translocation of a previously silent region to a position under control of a strong promoter. This mutation, which became fixed soon after its appearance, involved a deletion in the glutamate synthase operon that placed its upstream regulatory sequences in proximity to a non-genic region. The endpoints of this deletion show no appreciable sequence similarity to one another, indicating that it was the product of illegitimate recombination. This type of event is unusual in the LTEE, since most deletions in the LTEE arise from homologous recombination between repeat elements, including IS elements [50,51].

Translocations have previously been implicated in de novo gene birth and the origin of new functions, for example, by generating a new sRNA gene in *Salmonella* [52] and by providing an upstream promoter to the nascent AFGP antifreeze gene in codfish [53]. Although genomic rearrangements that lead to the formation of new genes are infrequent events, relocation of the regulatory region of expressed genes can immediately confer stable transcription and translation, as required for protein-coding gene birth. The large deletion in the glutamate synthase operon is the only case we observed in which the transition to a proto-gene was accompanied by translation. In contrast, we failed to detect any case in which an ancestrally transcribed region later acquired translation, which is in accordance with the overall rarity of changes in translation relative to changes in transcription of genes in the LTEE [36].

Aside from promoter recruitment, proto-genes were also generated by small deletions or point mutations, although in most cases, new promoters were not evident or recognizable. SNPs and small indels represent over 95% of mutations that we observed in the LTEE, so their relatively minor contribution to the emergence of proto-genes seems puzzling given that functional promoters are pervasive in sequence space [54,55] and occur frequently in bacterial genomes [56]. It is possible that promoters formed de novo are weaker than preexisting promoters, which have already been shaped by selection and regulatory mechanisms that prevent low levels of profligate transcription, such as H-NS [57], transcription termination factors [58], and genetic context [59]. As indicated by the fact that promoters supplied by IS*150* elements do not consistently secure downstream transcription, the recruitment of preexisting promoters is subject to some regulatory constraints despite being strong promoters. The one case of de novo promoter formation that led to strong expression was caused by a deletion-mediated repositioning of preexisting motifs (Fig 6B)—a consequence that would be impossible to achieve via SNPs or most small indels.

Given that there is widespread transcription throughout *E*. *coli* genomes [14,26], initial expression of non-genic regions may be common, at least stochastically in some cells in a population. LTEE-evolved cells have previously been reported to contain higher overall mRNA abundances compared to ancestors [36]. Our findings further indicate that even regions with very low initial expression in the ancestor have higher expression in evolved cells. For nascent transcripts to be retained by selection, expression needs to stably occur in different cells in a population, persist across evolutionary time, and reach a certain minimum threshold [38]. We demonstrate that when caused by regulatory mutations, non-genic expression can arise and persist [15,60,61].

Based on the total number of lines examined, we estimate the rate of proto-gene emergence to be about 1 event per 60,000 generations, with some appearing as early as 4,000 generations (Fig 9). To ensure that our final set of proto-genes were authentic and evolutionarily novel, we excluded all cases that did not have a readily identifiable causal mutation as well as those whose corresponding regions were expressed in the LTEE ancestor strain in a variety of different culture conditions. The RNA-seq and Ribo-seq datasets we used could not distinguish between overlapping transcripts or peptides produced from the same strand, so we could not investigate novel expression internal to annotated genes. Given the stringency and limitations of these criteria, our findings can be viewed as a minimal estimate of proto-gene emergence.

To date, most strain- and lineage-specific genes have been identified by comparative genomics, such that new ORFs represent either a transition from non-genic regions in closely related organisms or sequences gained through transfer from distantly related or unidentified taxa. In contrast, sequencing and transcriptomic data from the LTEE [35,36] permits the direct observation of proto-gene formation in individual lineages without horizontal transfer of genes from external sources. Genome-wide transcriptome surveys address one end of the de novo gene emergence puzzle, i.e., the availability of raw material for selection to act upon [12]. At the other extreme, functional screens of random peptides are able to assess whether stably translated peptides confer an adaptive benefit [62–64]. We adopted an intermediate approach that traced how the proto-genic raw material arises, and once available, whether it persists. Future experimental studies will be required to establish whether the novel transcripts and peptides we detected affect *E*. *coli* fitness or are byproducts of mutations that are beneficial because of their effects on nearby, existing genes. In either case, expression of novel RNA and protein proto-genes creates new opportunities for further evolution.

## Materials and methods

### LTEE strains

Strains were selected from the LTEE, which consists of 12 populations of *E*. *coli* that have been propagated in the laboratory since 1988 [65]. We examined transcription and/or translation in clonal isolates from: (i) 11 of the 12 LTEE populations (all but Ara+6) at 50,000 generations using existing datasets generated by others [36]; and (ii) the Ara−3 population at generations 5,000, 10,000, 15,000, 20,000, 25,000, 27,000, 30,000, 31,500, and 33,000 using new datasets generated for this study (S1 Fig and S1 Table). Most Ara−3 clones were selected because they possess sets of mutations that place them close to the lineage that evolved citrate utilization [66] (S1 Fig). All datasets include comparable information for their LTEE ancestor (REL606 and REL607 for Ara− and Ara+ strains, respectively).

### Transcriptomes

RNA-seq and Ribo-seq reads for the 50,000-generation clones were obtained from [36], and non-LTEE RNA-seq datasets for were acquired from [39,40]. The 50,000-generation clones each had 2 replicates for both RNA-seq and Ribo-seq. For the Ara−3 time series, RNA was isolated from 3 biological replicates of each strain cultured on separate days. For each replicate, we revived frozen stocks by inoculation into 10 ml of Davis Minimal media supplemented with 2 μg/l thiamine and 500 mg/l glucose (DM500). After overnight growth at 37°C, 500 μl of each culture was diluted into 50 ml of prewarmed DM500 and grown for an additional 24 h. Subsequently, 500 μl of these preconditioned cultures were inoculated into 50 ml DM500 and grown to 30% to 50% of the maximum observed OD_600_ at stationary phase. Cells were harvested by centrifugation, washed twice with saline, and flash-frozen on liquid nitrogen.

RNA was extracted from frozen cellular pellets using the RNASnap method [67]. Resulting supernatants were purified using Zymo Clean & Concentrator-25 columns (Zymo Research) incorporating the on-column DNase treatment step. The integrity of purified RNA was assessed with TapeStation (Agilent), and ribosomal RNAs were depleted using the gram-negative bacteria RiboZero rRNA Removal Kit (Epicentre). Final eluates were used as input for strand-specific RNA-seq library construction using the NEBNext RNA Library Prep Kit (New England Biolabs). Libraries were fractionated on 4% agarose E-gels (Invitrogen), and amplicons ranging from 0.2 to 8 kb were extracted and purified using the Zymoclean Gel DNA Recovery Kit (Zymo Research), quantified using a Qubit 2.0 fluorometer (Life Technologies), and stored at −80°C prior to sequencing. Libraries were sequenced on an Illumina HiSeq 4000 by the Genomic Sequencing and Analysis Facility (GSAF) at the University of Texas at Austin to generate 2 × 150-base paired-end reads. Raw FASTQ files of reads are available in the NCBI Sequence Read Archive (PRJNA896785).

### Data processing and analysis

For the non-LTEE datasets acquired from [39,40], raw reads were processed with Trimmomatic [68] by removing adapter sequences and low-quality bases from both ends, and only reads longer than 29 bases were retained. Reads from the 50,000-generation dataset were stripped of adaptor sequences, demultiplexed, dereplicated, end-trimmed based on read quality, and depleted of rRNAs using scripts available from [36]. The processed FASTQ files from all datasets were mapped to their respective genomes using Bowtie2 [69], using the “local” alignment option in “very sensitive” mode, with default values for all other parameters.

### Expression analysis

Assessing gene expression changes in the context of known genomic features requires mapping reads to a reference sequence. This process is complicated in LTEE strains by mutations that have added, deleted, and changed the coordinates of genomic features. For this and all subsequent analyses, we used lists of the mutations present in each LTEE clone that were compiled in prior whole-genome resequencing studies [38,50] and are available as GenomeDiff files in the LTEE-Ecoli genomic data repository (v2.0.1) [70]. Genome sequences of each evolved clone were generated using these GenomeDiff files and the *gdtools* APPLY command from *breseq* [71].

To estimate expression in both annotated and noncoding regions of the genome, we adopted a modified version of the method used by [14]. The ancestral genome was partitioned into 400-bp windows, which were searched against each evolved genome using GMAP to extract map coordinates [72]. To account for deletions, duplications, and spurious mapping, windows that either mapped more than once, had a >10 bp insertion or deletion, overlapped with an insertion sequence, or failed to map in any of the evolved genomes in either dataset were removed from the analysis. The final list of windows common to all time points in both datasets (*n* = 18,746) covered 81.2% of the genome on both strands. Windows that overlapped with annotated genes by more than 10 bp on the same strand were marked as “annotated,” and the remainder marked as “non-genic.” This latter category was further subdivided into “antisense” and “intergenic” windows, depending on whether they overlapped a coding gene on the opposite strand. Non-genic windows within the upstream 300 bp or downstream 100 bp of an annotated gene on the same strand were considered subject to transcription initiation before the start codon or to readthrough after the stop codon, respectively. Annotated genes were considered to correspond to the length of the coding sequence. Overlaps and distances between sequences were determined using the “intersect” and “closest” utilities in BEDTools [73]. The transcriptome-construction and window-categorization processes are summarized in S3 Fig.

For read counting and differential expression analysis, we generated separate annotation files for each LTEE clone by extracting the genome-specific coordinates of each window as informed by searches against evolved genomes using GMAP. Numbers of RNA-seq and Ribo-seq reads mapping to each feature in the annotation files were counted using the “htseq-count” tool from the HTSeq package [74]. In cases where a read mapped to more than one feature, the “nonunique-all” option of htseq-count assigned the read to count for all of these features.

Normalized read-counts expressed as mean transcripts per million (TPM) were calculated for each replicate and averaged as follows:

$$NRC\;(normalized\;read\;count)=\frac{read\;count\;per\;feature}{length\;of\;feature\;(BP)}$$

$$TPM\;(transcripts\;per\;million)=\left[\frac{NRC}{\sum \left(NRC\right)}\right]\times 10^{6}$$

Differential expression of corresponding windows in the ancestor and each evolved line was analyzed using the DESeq2 package in R [75] with apeglm normalization [76] and default parameters, with evolved and ancestral populations considered the treatment and control groups, respectively. A Wald test-generated *p*-value of 0.05 (adjusted by the Benjamini–Hochberg method) was used as the threshold for considering an element to be differentially expressed. To assess differential translation of windows while accounting for changes in transcription, we used the Riborex package [77] according to scripts provided in [36], with a *q*-value of 0.01 used as the threshold for significance.

### Proto-gene detection and characterization

To identify increases in transcription or translation associated with the appearance of new mutations in the 50,000-generation dataset, we extracted 100- and 200-bp regions immediately downstream of mutations from each ancestral and evolved genome, under the expectation that changes in expression would occur within this distance. For mutations other than those caused by IS*150* insertions, which have promoters oriented on a particular strand, regions were extracted from both strands. We then removed regions with >10-bp same-strand overlap with any annotated RNA gene, protein-coding gene, pseudogene, or repeat region with the “intersect” utility of BEDTools. To generate normalized read counts for later steps, we added in annotated gene coordinates to this list of sequences to construct our final transcriptome to identify proto-gene emergence. Read counting and differential expression analysis were conducted as described above.

All regions exhibiting statistically significant increases in transcription or translation relative to the ancestor were extracted and mapped back to their respective genomes with GMAP. Regions appearing more than once, cases where the adjacent mutations are counted twice by the breseq pipeline, and those that overlap with repeat regions on the opposite strand were removed, leaving a total of 63 and 29 regions with increased transcription and translation in the 50,000-generation dataset, respectively. For these regions, we generated transcription coverage plots, which were visually inspected for the presence of ancestral transcription. To accomplish this, we converted each bam file into a genome coverage file with the “genomecov” utility in BEDTools, extracted coverage information for each region of interest, and visualized them with the ggplot2 package in R [78]. Of the 63 initial cases of transcription increase, 38 were excluded as containing ancestral transcription, and another 6 were classified as “ambiguous” (S4 Table). All but one of the 29 cases of translation increase were excluded because of ancestral translation or mismatch between replicates, with the exception also qualifying as a case of novel transcription. Proto-genes were extracted from the time series data in an identical manner, with minor modifications on account of the paired-end dataset available for this series.

To determine if candidate regions exhibit transcription under conditions that differ from those in the LTEE, we leveraged 2 large RNA-seq datasets generated from the ancestral LTEE strain grown in 34 environmental conditions [39,40] comprising different carbon sources, salt concentrations, and conditions of nutrient starvation. RNA was extracted from cells grown to exponential and stationary phases in the presence of gluconate and lactate as carbon sources, 4 concentrations of sodium (5 mM to 300 mM), 10 concentrations of magnesium (0.08 mM to 400 mM), as well as 9 stages of glucose and glycerol growth and starvation spanning 3 h to 2 weeks.

After processing raw files, we produced read counts and coverage plots for the 19 proto-gene candidates in each of the 152 RNA-seq samples, as described above (S3 Fig). We also searched for occurrences of the candidate proto-genes in the K-12 MG1655 transcriptome reported in [41], which was assembled from 3,376 RNA-seq datasets deposited in the Sequence Read Archive [79]. We extracted the co-ordinates of all annotated and non-annotated transcripts from the associated supplementary tables, extracted their sequences, and used blastn [80] to query this database with proto-gene sequences.

All proto-genes passing these filters were checked for the presence of newly formed promoters with iPromoter-2L [43] and the Promoter calculator [44]. As evidence of potential translation, we searched for Ribo-seq reads within transcribed regions and constructed coverage plots as described above. To determine the occurrence and frequency of proto-gene-causing mutations in the LTEE, we used the mixed-population sequencing data generated from each preserved strain in the LTEE (separated by 500-generation intervals) from the first 60,000 generations [35]. After trimming the raw files with fastp [81], we searched each time point with *breseq* to identify mutations responsible for the proto-genes. We extracted mutation frequencies in each population from the output GenomeDiff files and visualized them with the ggplot2 package in R. Scripts used for analyses conducted in this study and numerical data underlying Figs 2, 4–9 and S2 are available at https://zenodo.org/records/10980486, DOI: 10.5281/zenodo.10980486.

## Supporting information

S1 File Windows displaying transcription, translation, and differential expression in 50,000-generation and time series datasets. All windows passing the cutoff in any of the corresponding replicates have been counted.
(XLSX)

S2 File Coverage plots of ancestral and evolved transcription in regions with upstream mutations that showed significantly higher expression in the 50,000-generation. Purple and orange lines represent ancestral and evolved transcription, respectively. Cases included in the final list of proto-genes are placed within orange boxes.
(PDF)

S3 File Coverage plots of ancestral and evolved translation in regions with upstream mutations that showed significantly higher expression in the 50,000-generation. Purple and orange lines represent ancestral and evolved translation, respectively. Cases included in the final list of proto-genes are placed within orange boxes.
(PDF)

S4 File Coverage plots of transcription in regions with upstream mutations that showed significantly higher expression in any of the evolved time points in the time series dataset. Cases included in the final list of proto-genes are placed within orange boxes.
(PDF)

S1 Table Strains used in this study. (XLSX)

S2 Table Mutation-linked novel transcription identified in both datasets. (XLSX)

S3 Table Open reading frames (ORF) contained in the proto-gene regions. Any ORF (>30 bp) found within the upstream 200 bp (with the ORF spanning the mutation) to the downstream 500 bp region of the proto-gene associated mutations are listed.
(XLSX)

S4 Table Mutations in non-genic regions investigated in the study. The number of novel transcription cases and proto-genes associated with each class of mutation is listed.
(XLSX)

S5 Table RNA counts of candidate proto-genes in non-LTEE expression library. Counts were calculated in 200 bp regions downstream of the mutation, except in 4 cases where non-genic up-regulation was only seen in the downstream 100 bp region.
(XLS)

S1 Fig Relationships among clones in the time series dataset. All but 2 clones cannot utilize citrate: ZDB564 has a rudimentary (Cit+) and CZB154 has a fully developed (Cit++) phenotype. Two clones (ZDB199, ZDB200) stem from highly diverged clades that did not evolve citrate utilization. Figure adapted from [66].
(TIF)

S2 Fig Coverage plots depicting transcription of candidate proto-genes in the non-LTEE expression library. The *y*-axis ranges are set according to the expression level of proto-gene candidates in the LTEE. Only cases having between 3 and 50 reads in at least 1 condition are shown. Each color represents level of transcription in 1 experiment in the library, and the 2 cases included in the final proto-gene list are placed within orange boxes. The data underlying this figure can be found in https://zenodo.org/records/10980486.
(EPS)

S3 Fig Transcriptome construction and categorization strategy for 400 bp windows in the 50,000-generation dataset. (EPS)
