# Supplementary figures and images for: Promoter recruitment drives the emergence of proto-genes in a long-term evolution experiment with Escherichia coli
Source: PLoS Biol. 2024 May 7;22(5):e3002418. doi: 10.1371/journal.pbio.3002418 (PMC11101190; doi:10.1371/journal.pbio.3002418)

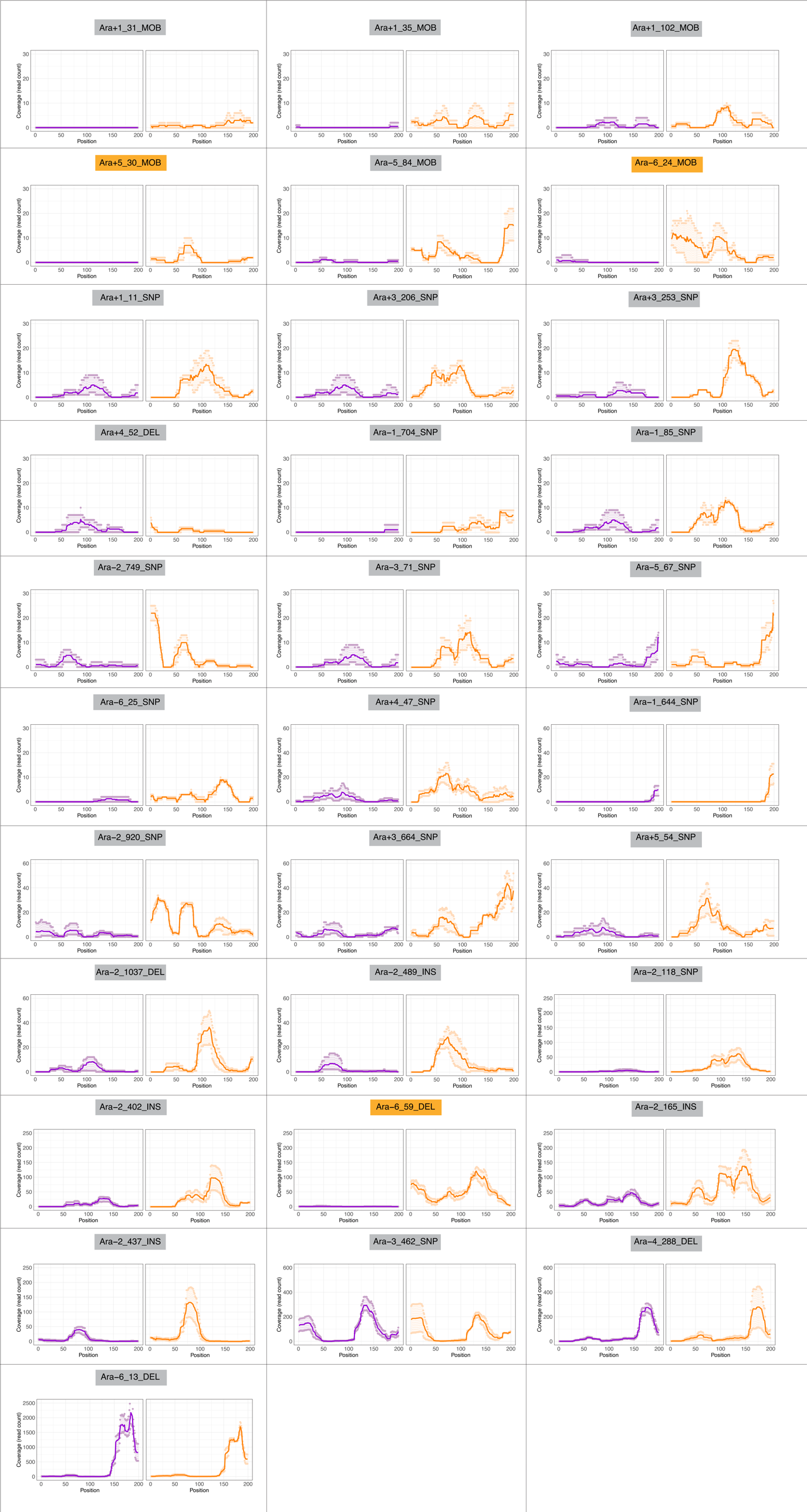

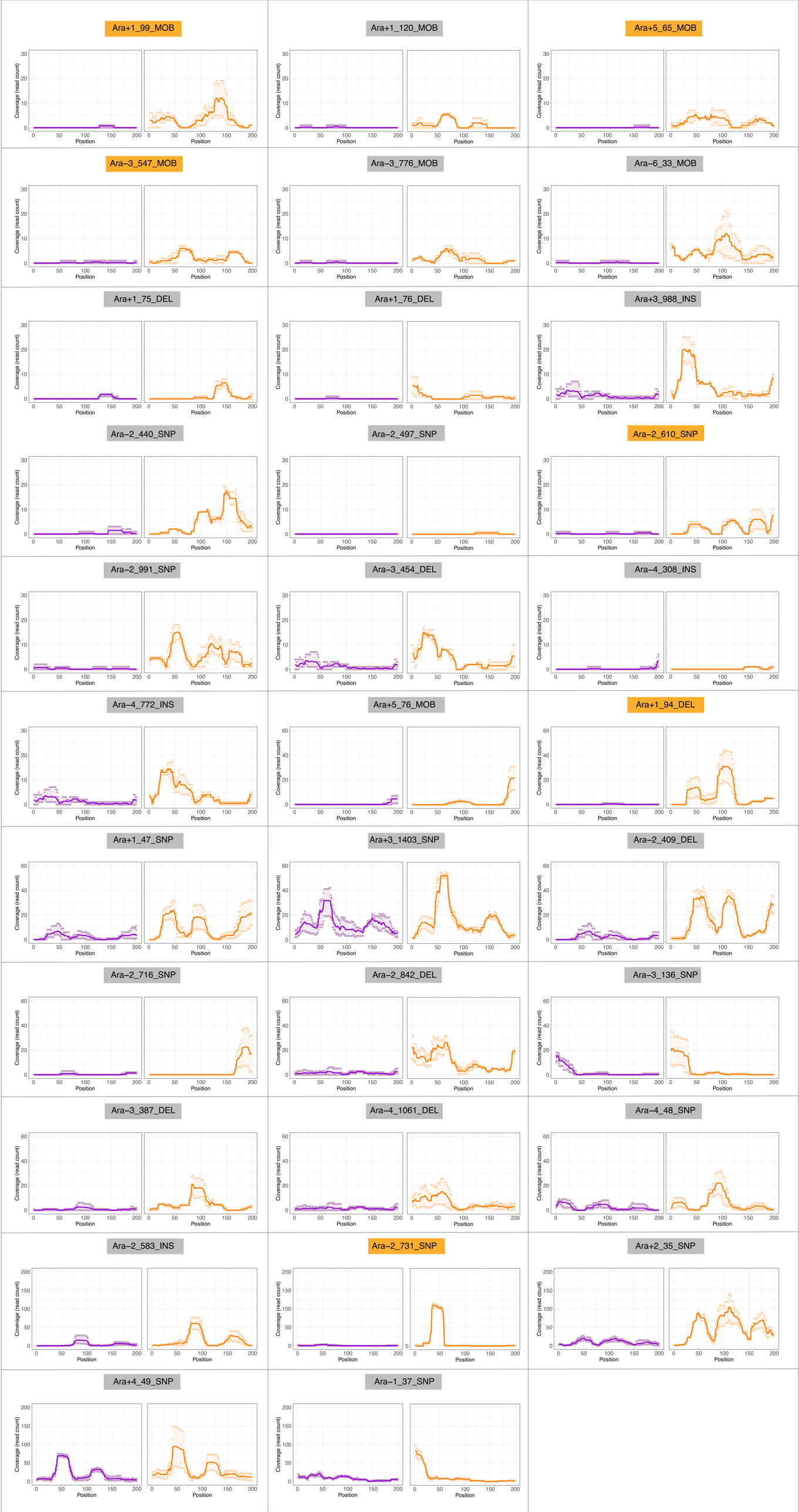

Supplement: S2 File — Purple and orange lines represent ancestral and evolved transcription, respectively. Cases included in the final list of proto-genes are placed within orange boxes. (PDF) [file pbio.3002418.s002.pdf]

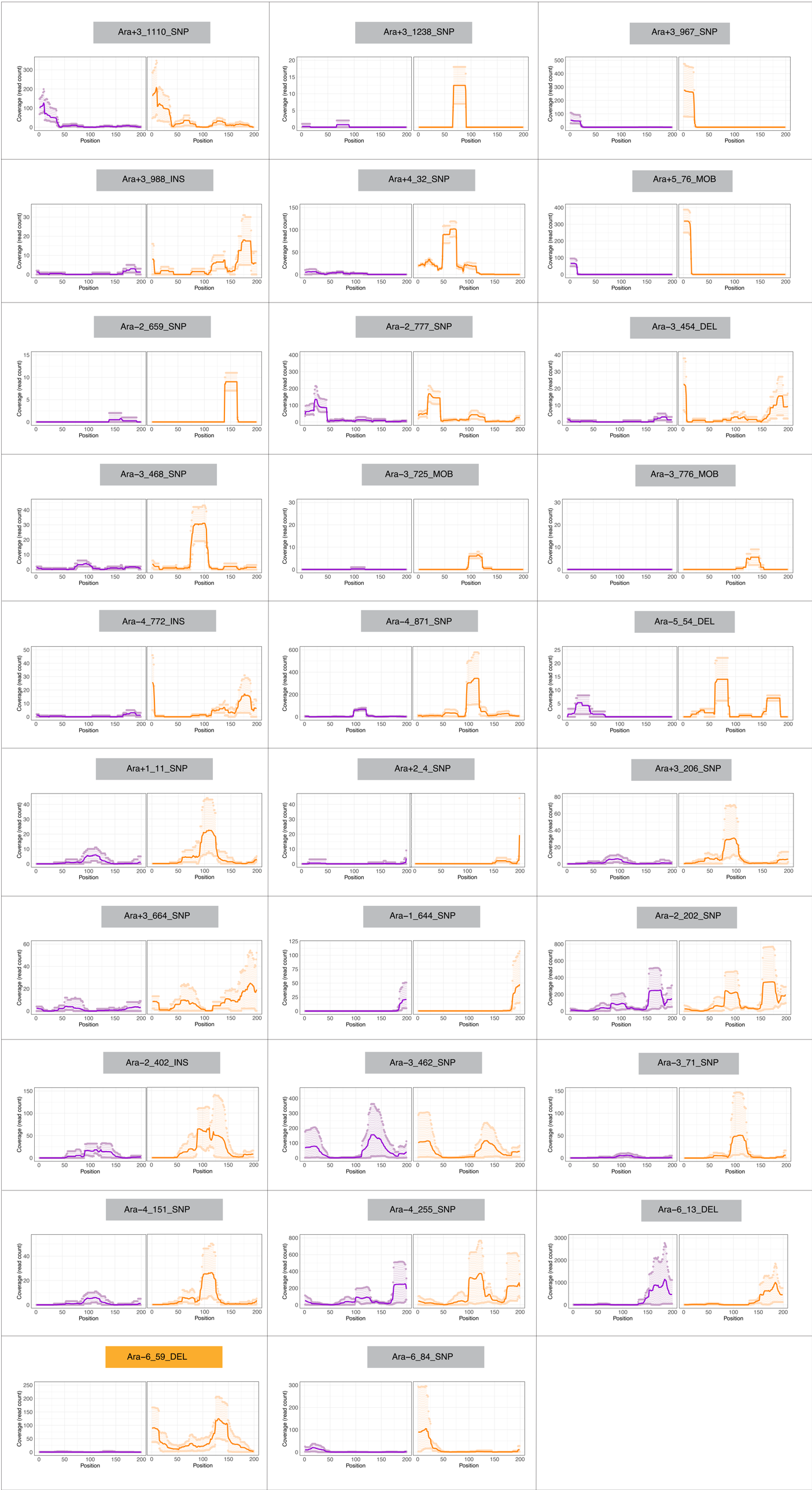

Supplement: S3 File — Purple and orange lines represent ancestral and evolved translation, respectively. Cases included in the final list of proto-genes are placed within orange boxes. (PDF) [file pbio.3002418.s003.pdf]

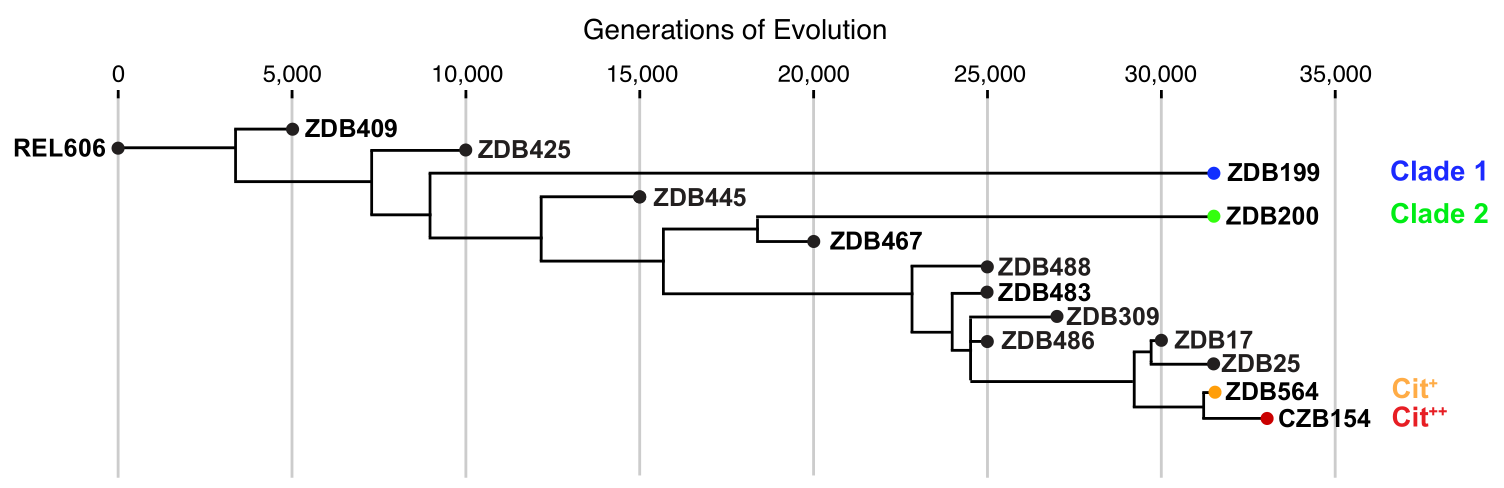

Supplement: S1 Fig — All but 2 clones cannot utilize citrate: ZDB564 has a rudimentary (Cit+) and CZB154 has a fully developed (Cit++) phenotype. Two clones (ZDB199, ZDB200) stem from highly diverged clades that did not evolve citrate utilization. Figure adapted from [66]. (TIF) [file pbio.3002418.s010.tif]
