# Supplementary material for: Promoter recruitment drives the emergence of proto-genes in a long-term evolution experiment with Escherichia coli
Source: PLoS Biol. 2024 May 7;22(5):e3002418. doi: 10.1371/journal.pbio.3002418 (PMC11101190; doi:10.1371/journal.pbio.3002418)
Supplement: S4 File — Cases included in the final list of proto-genes are placed within orange boxes. (PDF) [file pbio.3002418.s004.pdf]

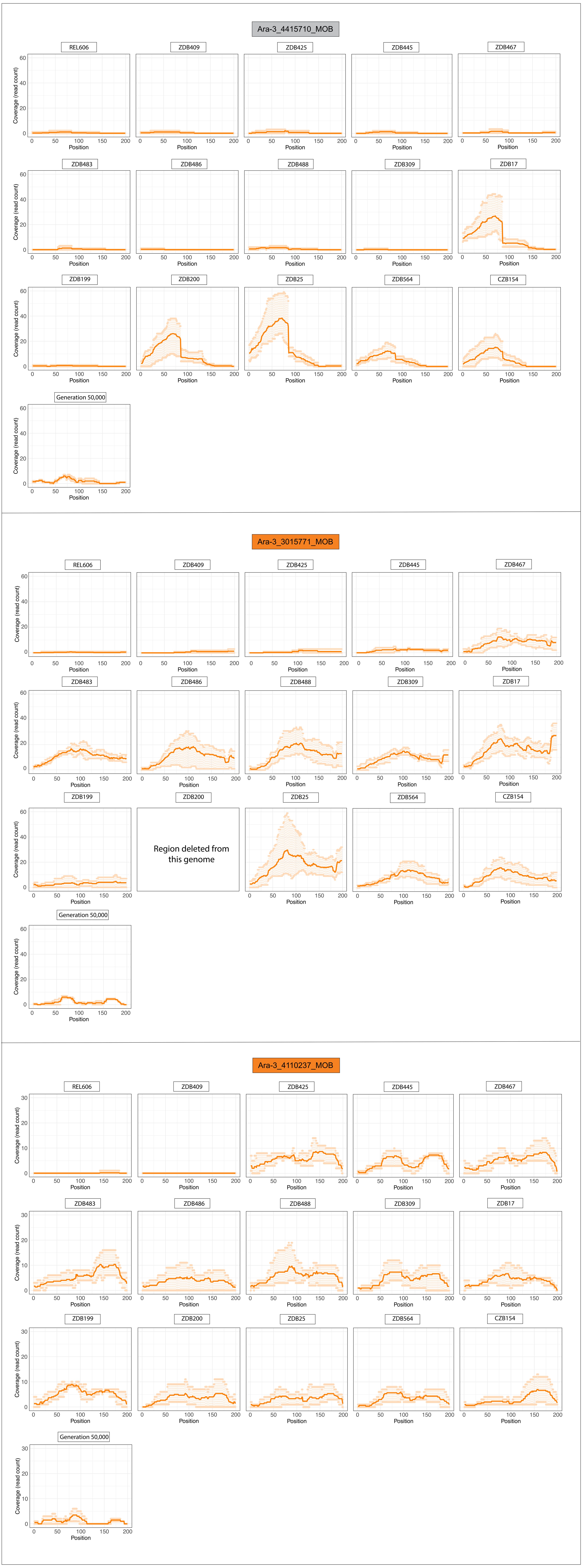

Ara-3_4415710_MOB
REL606
ZDB409
ZDB425
ZDB445
ZDB467
ZDB483
ZDB486
ZDB488
ZDB309
ZDB17
ZDB199
ZDB200
ZDB25
ZDB564
CZB154
Generation 50,000
Coverage (read count)
Position
Ara-3_3015771_MOB
REL606
ZDB409
ZDB425
ZDB445
ZDB467
ZDB483
ZDB486
ZDB488
ZDB309
ZDB17
ZDB199
ZDB200
Region deleted from this genome
ZDB25
ZDB564
CZB154
Generation 50,000
Coverage (read count)
Position
Ara-3_4110237_MOB
REL606
ZDB409
ZDB425
ZDB445
ZDB467
ZDB483
ZDB486
ZDB488
ZDB309
ZDB17
ZDB199
ZDB200
ZDB25
ZDB564
CZB154
Generation 50,000
Coverage (read count)
Position

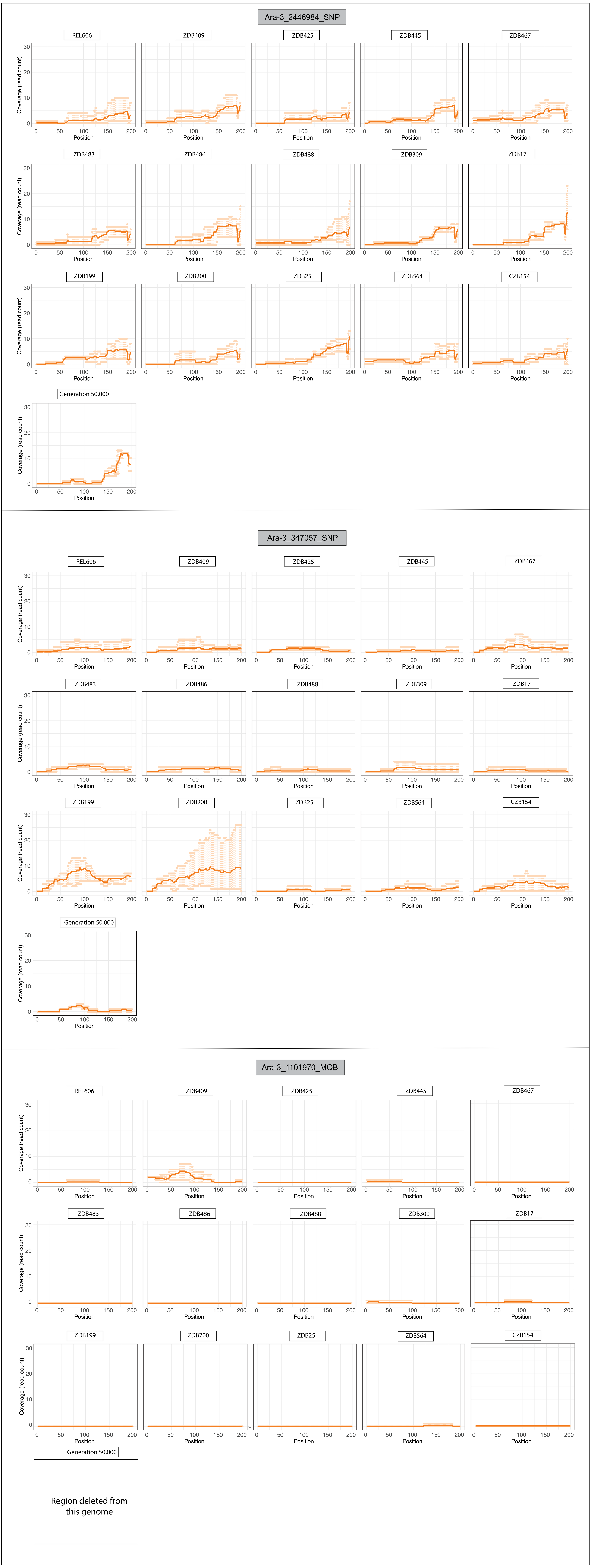

Ara-3_2446984_SNP
REL606
ZDB409
ZDB425
ZDB445
ZDB467
ZDB483
ZDB486
ZDB488
ZDB309
ZDB17
ZDB199
ZDB200
ZDB25
ZDB564
CZB154
Generation 50,000
Coverage (read count)
Position
Ara-3_347057_SNP
REL606
ZDB409
ZDB425
ZDB445
ZDB467
ZDB483
ZDB486
ZDB488
ZDB309
ZDB17
ZDB199
ZDB200
ZDB25
ZDB564
CZB154
Generation 50,000
Coverage (read count)
Position
Ara-3_1101970_MOB
REL606
ZDB409
ZDB425
ZDB445
ZDB467
ZDB483
ZDB486
ZDB488
ZDB309
ZDB17
ZDB199
ZDB200
ZDB25
ZDB564
CZB154
Generation 50,000
Region deleted from this genome
Coverage (read count)
Position

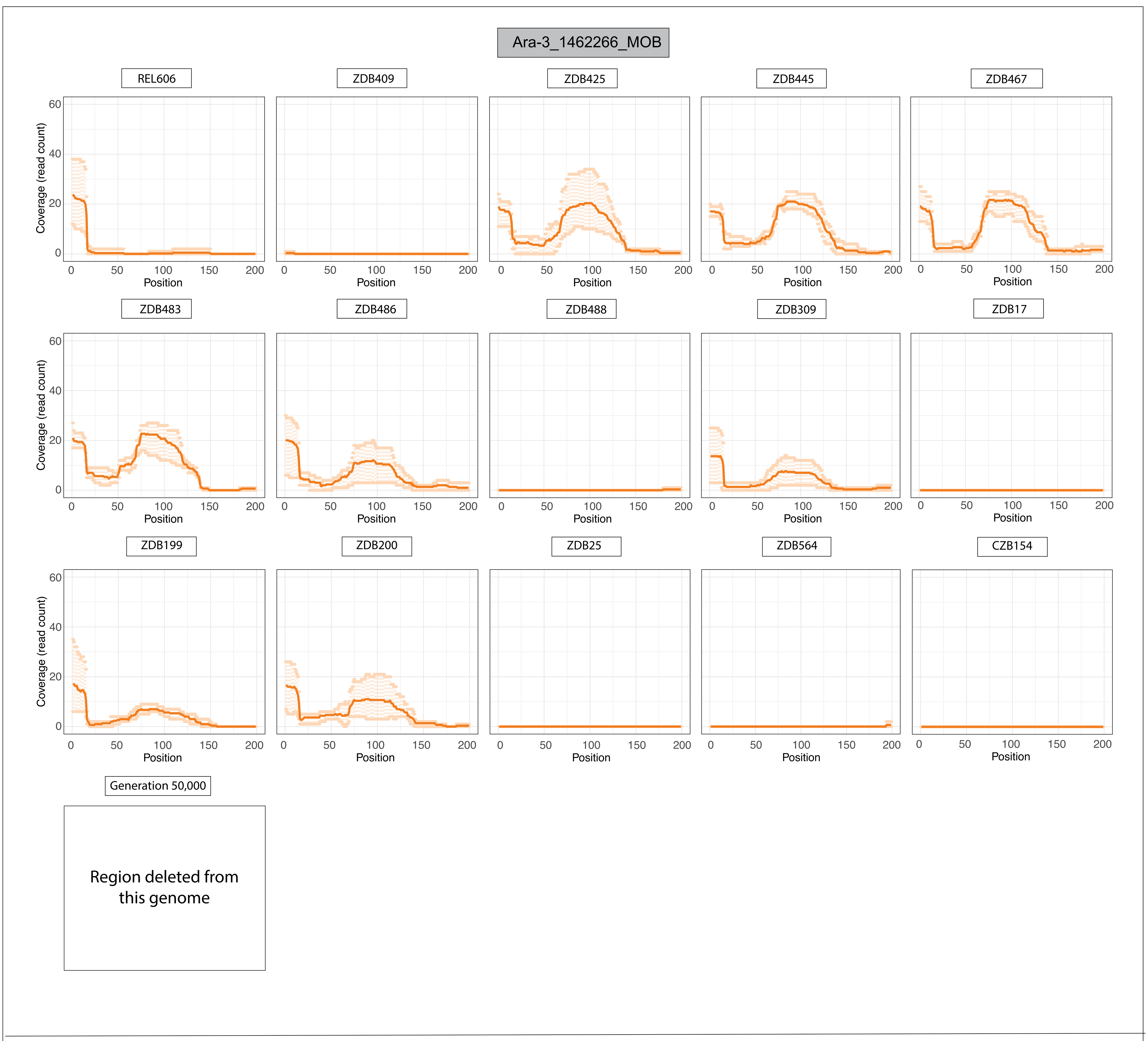
Ara-3_1462266_MOB
REL606
ZDB409
ZDB425
ZDB445
ZDB467
ZDB483
ZDB486
ZDB488
ZDB309
ZDB17
ZDB199
ZDB200
ZDB25
ZDB564
CZB154
Generation 50,000
Coverage (read count)
Position
Region deleted from this genome

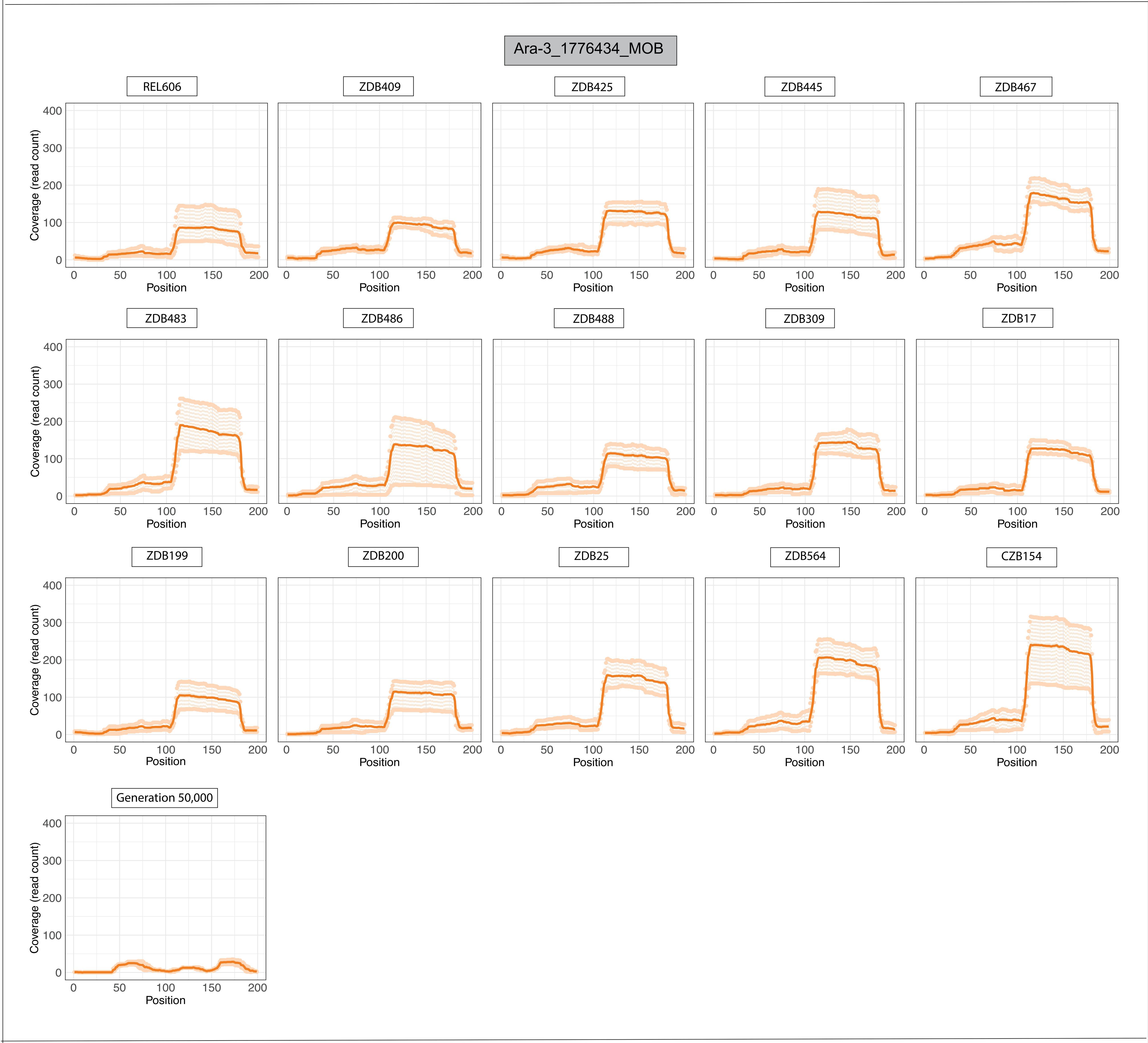
Ara-3_1776434_MOB
REL606
ZDB409
ZDB425
ZDB445
ZDB467
ZDB483
ZDB486
ZDB488
ZDB309
ZDB17
ZDB199
ZDB200
ZDB25
ZDB564
CZB154
Generation 50,000
Coverage (read count)
Position

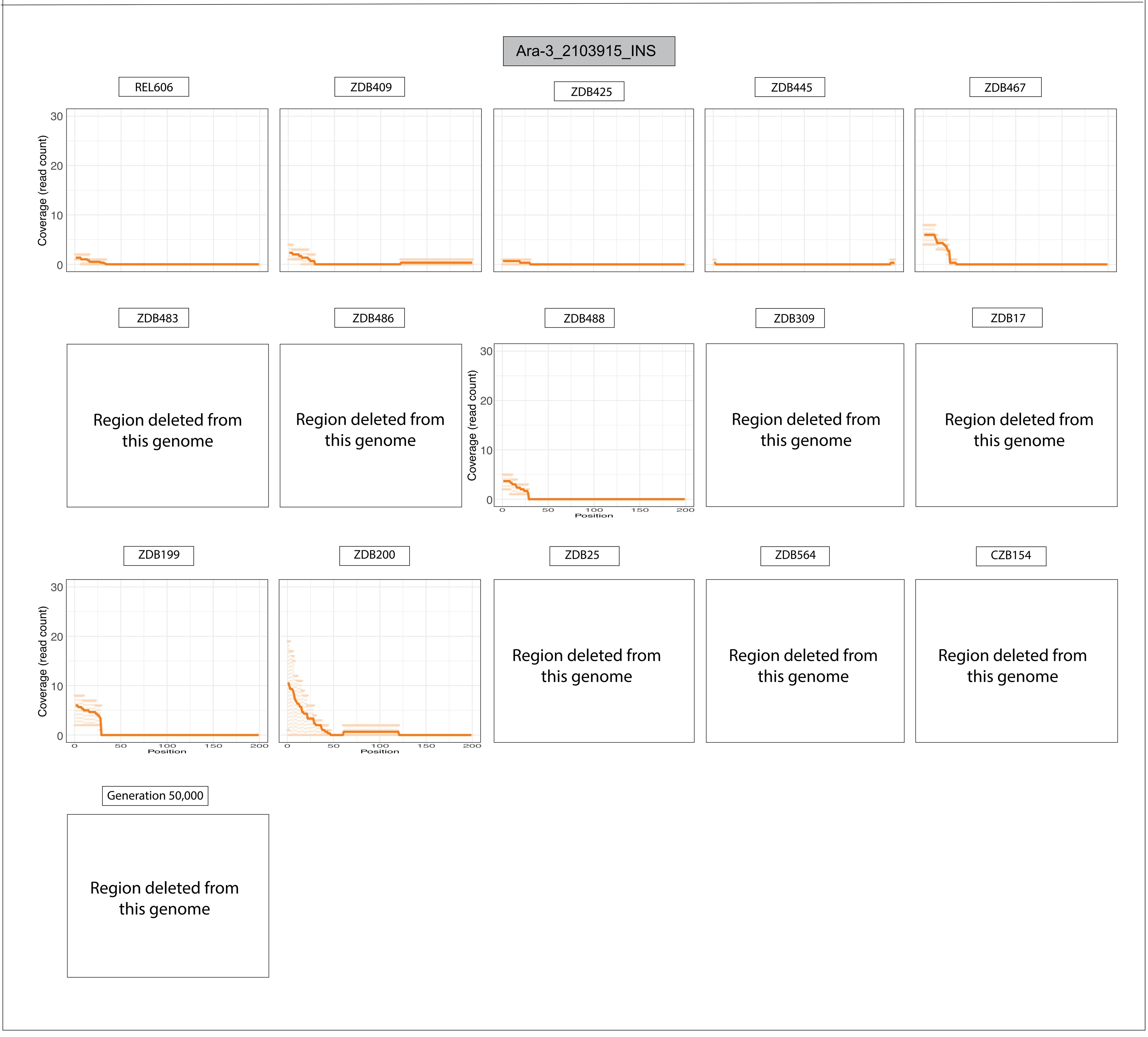
Ara-3_2103915_INS
REL606
ZDB409
ZDB425
ZDB445
ZDB467
ZDB483
ZDB486
ZDB488
ZDB309
ZDB17
ZDB199
ZDB200
ZDB25
ZDB564
CZB154
Generation 50,000
Coverage (read count)
Position
Region deleted from this genome
